# Multimodal nomogram for predicting axillary pathological complete response after neoadjuvant therapy in clinically node-positive breast cancer

**DOI:** 10.3389/fonc.2026.1758310

**Published:** 2026-04-10

**Authors:** Minyi Cheng, Jieqing Li, Yingyi Lin, Jiachen Zou, Dongqing Wang, Cangui Wu

**Affiliations:** 1 Department of Breast Cancer, Cancer Center, Guangdong Provincial People’s Hospital (Guangdong Academy of Medical Sciences), Southern Medical University, Guangzhou, China; 2 School of Medicine, South China University of Technology, Guangzhou, Guangdong, China

**Keywords:** breast neoplasms, lymph nodes, magnetic resonance imaging, neoadjuvant therapy, nomograms, ultrasonography

## Abstract

**Objectives:**

This study aims to create a model that combines ultrasonography (US), magnetic resonance imaging (MRI) examination, and clinicopathological features to predict the axillary pathological complete response (pCR) of patients with breast cancer (BC) who receive neoadjuvant therapy (NAT).

**Methods:**

This retrospective study included 600 patients with node-positive breast cancer who were eligible for enrollment (clinical stage cT1–4 and cN1-3) and received neoadjuvant therapy from January 2011 to January 2024. Before biopsy and neoadjuvant therapy, these patients underwent ultrasound (US) and MRI imaging of breast lesions and axillary lymph nodes (ALNs), and clinicopathological features were recorded before and after NAT. All imaging evaluations were independently performed by two experienced breast radiologists (with >10 years of experience), and discrepancies were resolved by consensus. Independent risk factors for predicting ALN status after NAT were identified by univariate and multivariate analyses. These independent risk factors were used for nomogram construction.

**Results:**

Univariate logistic regression analysis revealed that the maximum diameter of the breast lesions on MRI after NAT (p < 0.001), MRI ADC-value after NAT (p < 0.001), maximum and minimum diameter of the ALN on US after NAT (p < 0.001), the Ki67 level (p < 0.001), tumor grade 3 (p = 0.017), primary ALN stage cN 2 (p = 0.022), efficacy evaluation of the neoadjuvant therapy, pT stage, MP classification, HR, HER2, and the presence of the Hilum of the lymph gland were significantly associated with ALN pCR after NAT (p < 0.05). In the multivariate logistic regression analysis, ypT2 (p < 0.001), ypT3 (p = 0.007), HER2 (p < 0.001), response PR (p = 0.007), efficacy evaluation (SD/PD) (p = 0.010), and the presence of the Hilum of the lymph gland on US after NAT(p < 0.001) were considered independent predictors of ALN pCR after NAT. The area under the curve (AUC) of the nomogram was 0.934(95% CI: 0.913-0.960) in the training set and 0.908 (95% CI: 0.867-0.950) in the validation set, with a sensitivity of 82.0% and a specificity of 89.1% in the training set.

**Conclusion:**

Our noninvasive model based on US, MRI, and clinicopathological features can help accurately identify patients with ALN pCR after NAT and prevent unnecessary axillary lymph node dissection (ALND).

## Introduction

In an increasing number of breast cancer (BC) patients with axillary lymph node metastasis (ALNM), neoadjuvant chemotherapy (NAC) can reduce the disease stage, improve the operation rate, breast preservation rate, and axillary preservation rate, and provide prognostic information according to the response to NAC (1, 2). In axillary lymph node (ALN)-positive BC patients, the pCR rate after neoadjuvant therapy (NAT) was 24%-46% for breast lesions and 30%-49% for axillary lesions (3, 4). For patients with ALNM after NAT, previous studies reported a high false negative rate (FNR) of sentinel lymph node biopsy (SLNB), with FNR occurring in > 10% of patients in the Z1071 and the SENTINA trials (5, 6). ALND is still recommended for most patients with positive ALNs (7). However, for patients with an axillary pathological complete response (axillary pCR), ALND can be avoided to reduce risks such as lymphedema and arm pain (8, 9).

**Figure 1:**
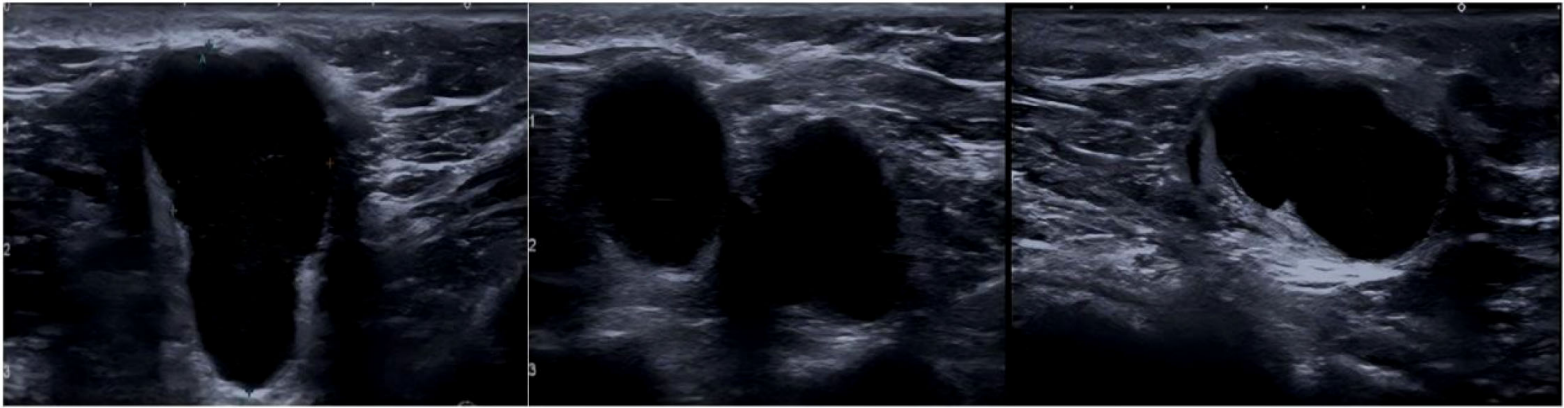
Axillary lymph nodes (LNs) show suspicious features at ultrasound (US), including cortical thickening more than 3 mm, round or irregular shape, and loss of fatty hilum at presentation.

**Figure 2:**
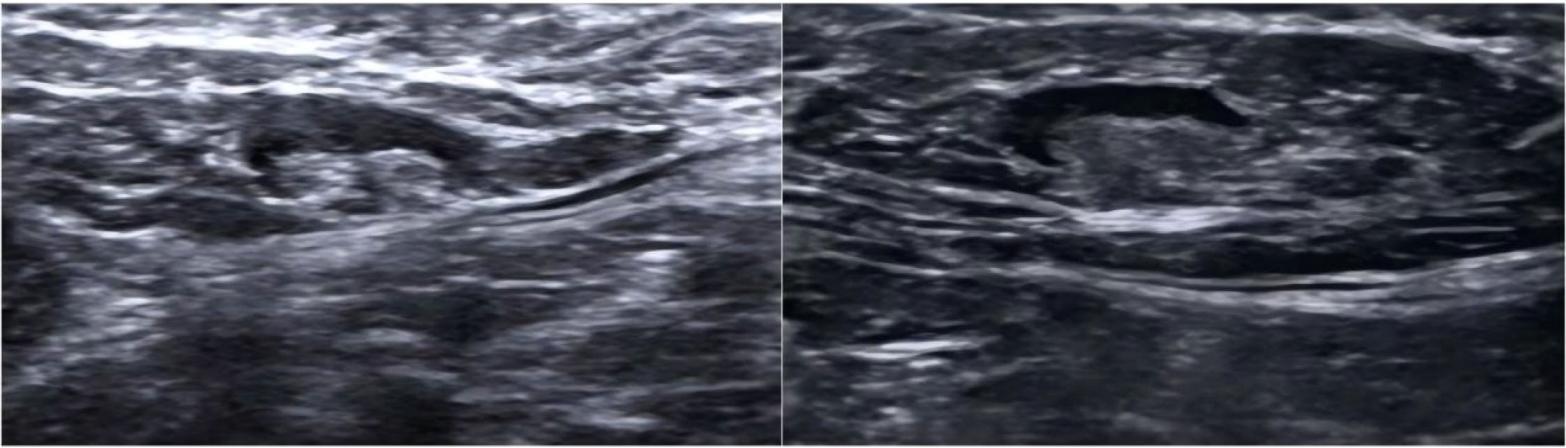
Normal appearance at ultrasound (US) after neoadjuvant therapy.

Breast imaging is commonly used to monitor the response to NAC, and in clinical practice, the axillary response is also tracked by MRI and US. According to earlier research, the sensitivity and specificity of ultrasound are 57–72% and 54–72%, respectively, whereas the sensitivity and specificity of MRI axillary imaging following NAT are 50–70% and 58–77%, respectively (10–13). Axillary ultrasound examination after NAC reduces the FNR of SLNB to 4–9.8% (14, 15). Monitoring the efficacy response during NAC helps identify responders and reduce the frequency of overtreatment, and nonresponders can be identified to allow changes in treatment to approaches that are more effective or early surgical intervention (16). For patients with BC undergoing NAT, ALN status is crucial and has been linked to disease-free survival (17–19). In this context, the use of axillary imaging to predict axillary pCR has become very important for helping surgeons choose axillary treatment after NAT. **Figure 1** shows the ultrasound images of lymph node metastasis before NAC. **Figure 2** shows the ultrasound images of a normal lymph node after NAC.

Several previous studies have developed nomograms to predict ALN pCR after NAT (20–24). However, these models have notable limitations: some relied solely on clinicopathological factors without incorporating imaging variables, while others focused on single imaging modalities. Kim et al. (2015) developed a nomogram using clinical variables alone, achieving an AUC of 0.82. Vila et al. (2016) incorporated clinical tumor response but did not include post-NAT ultrasound features. Our model advances beyond prior work by integrating multimodal imaging features (both MRI and ultrasound) with clinicopathological factors, achieving superior discriminatory performance (AUC: 0.934 in training, 0.908 in validation). Notably, we include the presence of the Hilum of the lymph gland on US after NAT, which has not been systematically evaluated in previous nomograms and provides important morphological information about lymph node response.

Accurate evaluation of axillary status is crucial for planning surgery for patients with ALNM BC undergoing NAT (25). Therefore, the purpose of this research was to develop a new nomogram based on clinicopathological factors and US and MRI imaging to predict post-NAT axillary status in patients with biopsy-proven node-positive BC after NAT, providing guidance for clinicians to develop objective and effective treatment options and avoid nonessential ALND.

## Materials and methods

### Patients and data collection

The Guangdong Provincial People’s Hospital Ethics Committee gave its approval for our retrospective study, which was carried out in compliance with the Declaration of Helsinki, and informed consent was waived (No. GDREC2019764H). We retrospectively analyzed 600 patients with primary ALNM BC in our hospital between January 2011 and January 2024. Eligible patients were defined as those with clinical stage cT1–4 and cN1–3 disease based on the American Joint Committee on Cancer (AJCC) 8th edition staging system. The inclusion criteria for patients were as follows: (1) underwent preoperative biopsy, MRI, and US; (2) ALNM confirmed by core needle biopsy; (3) underwent breast surgery and SLNB or ALND after NAT; and (4) received the current standard of treatment or under the center's clinical trial protocol, including doxorubicin plus paclitaxel, doxorubicin plus doxorubicin plus cyclophosphamide, doxorubicin plus cyclophosphamide plus paclitaxel, or HER-2 neu monoclonal antibody therapy. The exclusion criteria were as follows: (1) underwent biopsy or chemoradiotherapy before imaging; (2) clinical pathology data, US, or MRI data were incomplete; (3) did not undergo breast or axillary surgery after NAT; and (4) previous axillary malignancy or underwent axillary surgery. Patients with incomplete data (n=47) were excluded, and the potential selection bias from a single-center retrospective design was acknowledged as a limitation.

### Collection and analysis of clinicopathological and imaging features

Molecular subtype analysis of BC tissues was performed by immunohistochemistry (IHC). In accordance with the College of American Pathologists guidelines, estrogen receptor (ER), progesterone receptor (PR), Ki67, and human epidermal growth factor receptor 2 (HER-2) were detected. The surgical histopathological data included the number of metastatic lymph nodes, N stage, T stage, and histological grade (I, II, or III) as well as the expression of ER, PR, CerB-2, and Ki-67 in the histopathological report. ER- or PR-positive expression was greater than 1%, defined as hormone receptor positive(HR+), CerB-2 receptor 3+ or 2+ by IHC, and gene amplification was considered HER-2 positive (26, 27). The Miller–Payne (MP) grading system was used to evaluate pathological responses to NAT (28). Ki67 was analyzed as a continuous variable in the statistical analysis.

### Image interpretation

Retrospective analysis was done using ultrasound and MR images of ALN lesions and breast lesions taken before and after NAT. All imaging examinations were performed 2–4 weeks before and 2–4 weeks after the completion of NAT. All US and MRI images were independently evaluated by two experienced breast radiologists (Y.L. and J.Z., with >10 years of experience in breast imaging). In cases of disagreement, a consensus was reached through discussion. Inter-observer agreement was assessed using Cohen's kappa statistics, which showed substantial agreement (κ = 0.85 for US hilum assessment, κ = 0.88 for MRI measurements). The LN index was defined as the LN with the most possible metastatic characteristics and the highest cortical thickness if there were two or more ALNs. The radiographic characteristics of the lymph nodes were evaluated as follows: the shortest and the longest diameter of the LN (measured as the maximum longitudinal diameter [LNL] and maximum transverse diameter [LNS]) and the hilum of the lymph gland (presence: defined as a visible echogenic central hilum; absence: defined as complete loss or replacement of the echogenic hilum by hypoechoic cortical tissue) after NAT. This classification was based on established criteria for sonographic lymph node assessment, where a normal lymph node is characterized by a discernible central fatty hilum, and the absence of the hilum is considered a suspicious feature for malignancy (29). The maximum diameter of the breast lesions and apparent diffusion coefficient (ADC) on the MR images were collected before and after treatment.

### Evaluating the safety and efficacy of NAT

Four categories of response to NAT are distinguished by the Response Evaluation Criteria in Solid Tumors (RECIST) (version 1.1): complete response (CR), partial response (PR), progressing disease (PD), and stable disease (SD). MRI was used to evaluate the main tumor's clinical response in accordance with the Solid Tumors Response Evaluation Criteria (30). After NAT, CR was defined as the lack of palpable carcinoma and/or no visible carcinoma. A reduction of at least 30% in the size of the lesion or lesions was considered a partial response. A 20% rise in the size of the lesion or lesions was considered progressive disease (PD). When neither the PR nor the PD criteria were satisfied, stable disease was suggested.

### Data and statistical analysis

We randomly assigned the samples into a training and a validation set in a 7:3 ratio. For these sets, we performed between-group comparisons using either the chi-square test or Fisher's exact test (for categorical variables) and the independent samples t-test (for continuous variables). Based on the training set data, we conducted univariate logistic regression to evaluate the effects of the following factors on ALN pCR: age, primary pathology type, histological grade, clinical tumor (cT) stage, clinical node (cN) stage, the clinical response to NAT, the MP grading system, hormone receptor (HR) status, HER2 status, Ki67 index, and ypT; the LNs were evaluated as follows: transverse and longitudinal diameter and the hilum of the lymph gland (normal, without, or displaced) after NAT on the US. The maximum diameter of the breast lesions and the apparent diffusion coefficient (ADC) on the MR images collected before and after treatment. Significant variables from the univariate analysis were included in the multivariate logistic regression model (with a P-value < 0.05, to minimize the risk of overfitting while retaining clinically meaningful predictors) to construct a predictive model, which was visualized using a nomogram. The model’s discriminatory ability was assessed with receiver operating characteristic (ROC) curves and validated using the validation set. Calibration plots were used to assess the consistency between the predicted probabilities of ALN conversion and the actual results in ALN conversion cases in the validation set. We also used the Hosmer-Lemeshow test to evaluate the model’s goodness of fit (GOF). Additionally, the DeLong method was used to compare AUC differences between the training and validation sets, and the Bootstrap method was used to compute accuracy, sensitivity, specificity, positive predictive value (PPV), and negative predictive value (NPV) at the cutoff value corresponding to the maximum Youden index. All statistical analyses were conducted using R version 4.3.3 (Vienna, Austria; http://www.R-project.org). The significance level was set at α = 0.05.

## Results

### Patient characteristics

The study retrospectively enrolled 600 patients with ALNM BC who had received NAT and surgical treatment. A total of 327 (54.5%) patients achieved axillary pCR, and 273 (45.5%) had ALNM after NAC. The training set was as follows: 228/420 (54.3%) pCR, 192 (45.7%) residual. The validation set was as follows: 99/180 (55.0%) pCR, 81 (45.0%) residual. All the enrolled cases were divided into a training set of 420 cases and a validation set of 180 cases in a 7:3 ratio randomly. All the baseline features for both sets are described in **Table 1**.

**Table 1:** Characteristics of patients in the training and validation sets.

Characteristics	Training set (420)	Validation set (180)	P value
age,y(mean ± SD)	48.98 (10.31)	47.89 (9.67)	0.225
MRIpreT mm (mean ± SD)	32.25 (16.96)	31.27 (19.60)	0.537
MRIposT mm (mean ± SD)	13.39 (11.80)	13.41 (13.98)	0.986
preADC (mean ± SD)*10^-3^	0.89 (0.20)	0.87 (0.51)	0.314
posADC (mean ± SD)*10^-3^	1.24 (0.36)	1.24 (0.37)	0.962
preLNL mm (mean ± SD)	19.18 (9.48)	19.74 (9.80)	0.510
posLNL mm (mean ± SD)	11.47 (6.24)	10.63 (4.85)	0.108
preLNS mm (mean ± SD)	10.32 (5.18)	10.54 (5.50)	0.628
posLNS mm (mean ± SD)	5.84 (3.09)	5.63 (2.46)	0.434
Ki67 % (mean ± SD)	44.65 (23.21)	44.66 (22.71)	0.997
Primary pathological type			
IDC	409 (97.4)	172 (95.6)	0.360
ILC or other	11 (2.6)	8 (4.4)	
Histological grade			0.439
2	296 (56.2)	108 (60.0)	
3	184 (43.8)	72 (40.0)	
cT			0.251
T1	41 (9.8)	27 (15.0)	
T2	272 (64.8)	108 (60.0)	
T3	91 (21.6)	36 (20.0)	
T4	16 (3.8)	9 (5.0)	
cT			0.251
1	337 (80.2)	145 (80.6)	
2	71 (16.9)	27 (15.0)	
3	12 (2.9)	8 (4.4)	
Response			0.158
CR	153 (36.4)	70 (38.9)	
PR	245 (58.4)	94 (52.2)	
SD/PD	22 (5.2)	16 (8.9)	
MP			0.522
1	36 (8.5)	14 (7.8)	
2	44 (10.5)	20 (11.1)	
3	113 (26.9)	37 (20.6)	
4	57 (13.6)	29 (16.1)	
5	170 (40.5)	80 (44.4)	
ypT			0.511
1	181 (43.1)	79 (43.9)	
2	180 (42.9)	70 (38.9)	
3/4	59 (14.0)	31 (17.2)	
HR			0.386
positive	283 (67.4)	114 (63.3)	
negative	137 (32.6)	66 (36.7)	
HER2			
positive	187 (44.5)	76 (42.2)	0.667
negative	233 (55.5)	104 (57.8)	
Hilum of lymph gland			
existence	299 (71.2)	130 (72.2)	0.875
absence	121 (28.8)	50 (27.8)	

MRIpre T and MRIpos T, the maximum diameter of the breast lesions on the magnetic resonance (MR) images collected before and after treatment; LNL, longitudinal diameter of lymph node (maximum long axis); LNS, transverse diameter of lymph node (short axis); preLNL, posLNL, posLNS, and preLNS, the LNs were evaluated as follows: transverse and longitudinal on the ultrasound (US) diameter collected before and after treatment. preADC and posADC, apparent diffusion coefficient (ADC) on the MR images collected before and after treatment; IDC, Invasive Ductal Carcinoma; ILC, Invasive Lobular Carcinoma; cT, clinical tumor stage; cN, clinical node stage; MP, Miller-Payne; CR, Complete Response; PR, Partial Response; SD, Stable Disease; PD, Progressive Disease; HR, hormone receptor; ypT, Pathological T stage after neoadjuvant therapy.

### Selection of factors for constructing the model

In the univariate analysis, the variables of multivariate logistic regression analysis were age, primary pathology type, histological grade, cT stage, cN stage, response to NAT, MP grade, HR status, HER-2 status, Ki67 index, and ypT. The univariate logistic regression analysis revealed that the maximum diameter of the breast lesions on the MR images after NAT (p < 0.001), post ADC-value (p < 0.001), long and short diameter of the ALN after NAT on the US (p < 0.001), Ki67 level (p < 0.001), tumor grade 3 (p = 0.017), primary lymph node stage cN 2 (p = 0.022), efficacy evaluation of neoadjuvant therapy, pT by stage, MP grade, HR status, HER2 status, and the presence of the Hilum of the lymph gland on US after NAT were significantly associated with ALN pCR. In the multivariate logistic regression analysis, ypT 2 (p < 0.001), ypT 3 (p = 0.007), HER 2 (p < 0.001), response PR (p = 0.007), response SD/PD (p = 0.010), and the presence of the Hilum of the lymph gland on US after NAT(p < 0.001) were considered independent predictors of ALN pCR after NAT. These results are shown in **Tables 2**–**4**.

**Table 2:** Clinicopathologic characteristics of the patients who had positive lymph nodes (LNs) and negative LNs in the total enrolled population.

Characteristics	Negative LNs, n (%) ypN0/327	Positive LNs, n (%) ypN+/273	P value
age,y (mean ± SD)	48.53 (9.81)	48.80 (10.51)	0.745
MRIpreT mm (mean ± SD)	31.50 (17.45)	32.50 (18.18)	0.496
MRIposT mm (mean ± SD)	10.92 (11.30)	16.35 (13.19)	<0.001
preADC (mean ± SD)*10-3	0.89 (0.20)	0.88 (0.21)	0.315
posADC (mean ± SD)*10-3	1.32 (0.36)	1.14 (0.34)	<0.001
preLNL mm (mean ± SD)	18.75 (9.68)	20.05 (9.41)	0.097
posLNL mm (mean ± SD)	10.33 (5.18)	12.29 (6.45)	<0.001
preLNS mm (mean ± SD)	10.02 (5.34)	10.82 (5.18)	0.065
posLNS mm (mean ± SD)	5.29 (2.43)	6.36 (3.32)	<0.001
Ki67 % (mean ± SD)	48.44 (23.20)	40.11 (22.05)	<0.001
Primary pathological type			0.181
IDC	320 (97.9)	261 (95.6)	
ILC or other	7 ( 2.1)	12 (4.4)	
Histological grade			
2	173 (52.9)	171 (62.6)	0.020
3	154 (47.1)	102 (37.4)	
cT			0.875
T1	38 (11.6)	30 (11.0)	
T2	208 (63.6)	172 (63.0)	
T3	66 (20.2)	61 (22.3)	
T4	15 ( 4.6)	10 ( 3.7)	
cN			0.027
1	275 (84.1)	207 (75.8)	
2	45 (13.8)	53 (19.4)	
3	7 ( 2.1)	13 ( 4.8)	
Response	204 (62.4)		<0.001
CR	204 (62.4)	19 ( 7.0)	
PR	117 (35.8)	222 (81.3)	
SD/PD	6 ( 1.8)	32 (11.7)	
MP			<0.001
1	13 ( 4.0)	37 (13.6)	
2	15 ( 4.6)	49 (17.9)	
3	38 (11.6)	112 (41.0)	
4	37 (11.3)	49 (17.9)	
5	224 (68.5)	26 ( 9.5)	
ypT			<0.001
0/tis	227 (69.4)	33 (12.1)	
1	81 (24.8)	169 (61.9)	
≥2	19 ( 5.8)	71 (26.0)	
HR			<0.001
positive	186 (56.9)	211 (77.3)	
negative	141 (43.1)	62 (22.7)	
HER2			<0.001
positive	198 (60.6)	65 (23.8)	
negative	129 (39.4)	208 (76.2)	
Hilum of lymph gland			<0.001
existence	316 (96.6)	113 (41.4)	
absence	11 (3.4)	160 (58.6)	

MRIpre T and MRIpos T, the maximum diameter of the breast lesions on the magnetic resonance (MR) images collected before and after treatment; preLNL, posLNL, posLNS, and preLNS, the LNs were evaluated as follows: transverse and longitudinal on the ultrasound (US) diameter collected before and after treatment. preADC and posADC, apparent diffusion coefficient (ADC) on the MR images collected before and after treatment; IDC, Invasive Ductal Carcinoma; ILC, Invasive Lobular Carcinoma; cT, clinical tumor stage; cN, clinical node stage; MP, Miller-Payne; CR, Complete Response; PR, Partial Response; SD, Stable Disease; PD, Progressive Disease; HR, hormone receptor; ypT, Pathological T stage after neoadjuvant therapy.

**Table 3:** Univariate analysis of clinicopathologic characteristics of the patients who had positive lymph nodes (LNs) and negative LNs in the training set.

Characteristics	Negative LNs, n(%) ypN-	Positive LNs, n(%) ypN+	OR (95% CI)	P value
age,y(mean ± SD)	49.06 (9.94)	48.89 (10.76)	1.00 (0.98-1.02)	0.866
MRIpreT mm(mean ± SD)	32.10 (16.32)	32.43 (17.73)	1.00 (0.99-1.01)	0.844
MRIposT mm(mean ± SD)	10.84 (10.76)	16.41 (12.28)	0.96 (0.94-0.98)	**<0.001**
preADC(mean ± SD)*10^-3^	0.89 (0.18)	0.89 (0.21)	1.04 (0.39-2.77)	0.935
posADC(mean ± SD)*10^-3^	1.32 (0.36)	1.15 (0.33)	3.98 (2.24-7.08)	**<0.001**
preLNL mm(mean ± SD)	18.50 (9.67)	19.98 (9.21)	0.98 (0.96-1.00)	0.113
posLNL mm(mean ± SD)	10.41 (5.33)	12.73 (6.99)	0.94 (0.91-0.97)	**<0.001**
preLNS mm(mean ± SD)	9.99 (5.44)	10.71 (4.84)	0.97 (0.94-1.01)	0.158
posLNS mm(mean ± SD)	5.35 (2.58)	6.42 (3.53)	0.88 (0.81-0.95)	**<0.001**
Ki67 %(mean ± SD)	48.45 (23.43)	40.14 (22.16)	1.02 (1.01-1.02)	**<0.001**
Primary pathological type				
IDC	223(97.8)	186(96.9)	Ref	
ILC or other	5 ( 2.2)	6 ( 3.1)	0.70 (0.21-2.31)	0.553
Histological grade				
2	116 (50.9)	120 (62.5)	Ref	
3	112 (49.1)	72 (37.5)	1.61 (1.09-2.38)	**0.017**
cT				
T1	22 ( 9.6)	19 ( 9.9)	Ref	
T2	151 (66.3)	121 (63.1)	1.08 (0.56-2.08)	0.824
T3	46 (20.2)	45 (23.4)	0.88 (0.42-1.85)	0.741
T4	9 ( 3.9)	7 ( 3.6)	1.11 (0.35-3.55)	0.860
cN				
1	193 (84.6)	144 (75.0)	Ref	
2	30 (13.2)	41 (21.4)	0.55 (0.33-0.92)	**0.022**
3	5 ( 2.2)	7 ( 3.6)	0.53 (0.17-1.71)	0.291
Response				
CR	141 (61.8)	12 ( 6.2)	Ref	
PR	84 (36.7)	161 (83.9)	0.04 (0.02-0.08)	**<0.001**
SD/PD	3 ( 1.3)	19 ( 9.9)	0.01 (0.00-0.05)	**<0.001**
MP				
1	10 ( 4.4)	26 (13.5)	Ref	
2	11 ( 4.8)	33 (17.2)	0.87 (0.32-2.35)	0.779
3	26 (11.4)	87 (45.3)	0.78 (0.33-1.82)	0.561
4	27 (11.8)	30 (15.6)	2.34 (0.96-5.73)	0.063
5	154 (67.6)	16 ( 8.4)	25.02 (10.25-61.10)	**<0.001**
ypT				
0/tis	158 (69.3)	23 (12.0)	Ref	
1	57 (25.0)	123 (64.0)	0.07 (0.04-0.12)	**<0.001**
≥2	13 ( 5.7)	46 (24.0)	0.04 (0.02-0.09)	**<0.001**
HR				
negative	93 (40.8)	44 (22.9)	Ref	
positive	135 (59.2)	148 (77.1)	0.43 (0.28-0.66)	**<0.001**
HER2				
negative	85 (37.3)	148 (77.1)	Ref	
positive	143 (62.7)	44 (22.9)	5.66 (3.68-8.70)	**<0.001**
Hilum of lymph gland				
absence	9(3.9)	112(58.3)	Ref	
existence	219 (96.1)	80 (41.7)	34.07 (16.49-70.39)	**<0.001**

MRIpre T and MRIpos T, the maximum diameter of the breast lesions on the magnetic resonance (MR) images collected before and after treatment; preLNL, posLNL, posLNS, and preLNS, the LNs were evaluated as follows: transverse and longitudinal on the ultrasound (US) diameter collected before and after treatment. preADC and posADC, apparent diffusion coefficient (ADC) on the MR images collected before and after treatment; IDC, Invasive Ductal Carcinoma; ILC, Invasive Lobular Carcinoma; cT, clinical tumor stage; cN, clinical node stage; MP, Miller-Payne; CR, Complete Response; PR, Partial Response; SD, Stable Disease; PD, Progressive Disease; HR, hormone receptor; ypT, Pathological T stage after neoadjuvant therapy. Bold values represent variables with statistically significant differences (P < 0.05).

**Table 4:** Details of the prediction model.

Intercept and variable	Prediction model		
	Coefficient	Odds ratio (95% CI)	P
Intercept	0.928	–	**0.093**
ypT1	2.133	8.44 (2.98-23.89)	**<0.001**
ypT≥2	1.730	5.64 (1.62-19.63)	**0.006**
HER2	-1.204	0.30 (0.15-0.59)	**<0.001**
Hilum of lymph gland	-4.092	0.02 (0.01-0.05)	**<0.001**
Response2 (PR)	1.551	4.72 (1.52-14.67)	**0.007**
Response3 (SD/PD)	2.487	12.03 (1.80-80.39)	**0.010**

PR, Partial Response; SD, Stable Disease; PD, Progressive Disease; ypT, Pathological T stage after neoadjuvant therapy. Bold values represent variables with statistically significant differences (P < 0.05).

### Predictive nomogram for the probability of axillary pCR after NAT

**Figure 3:**
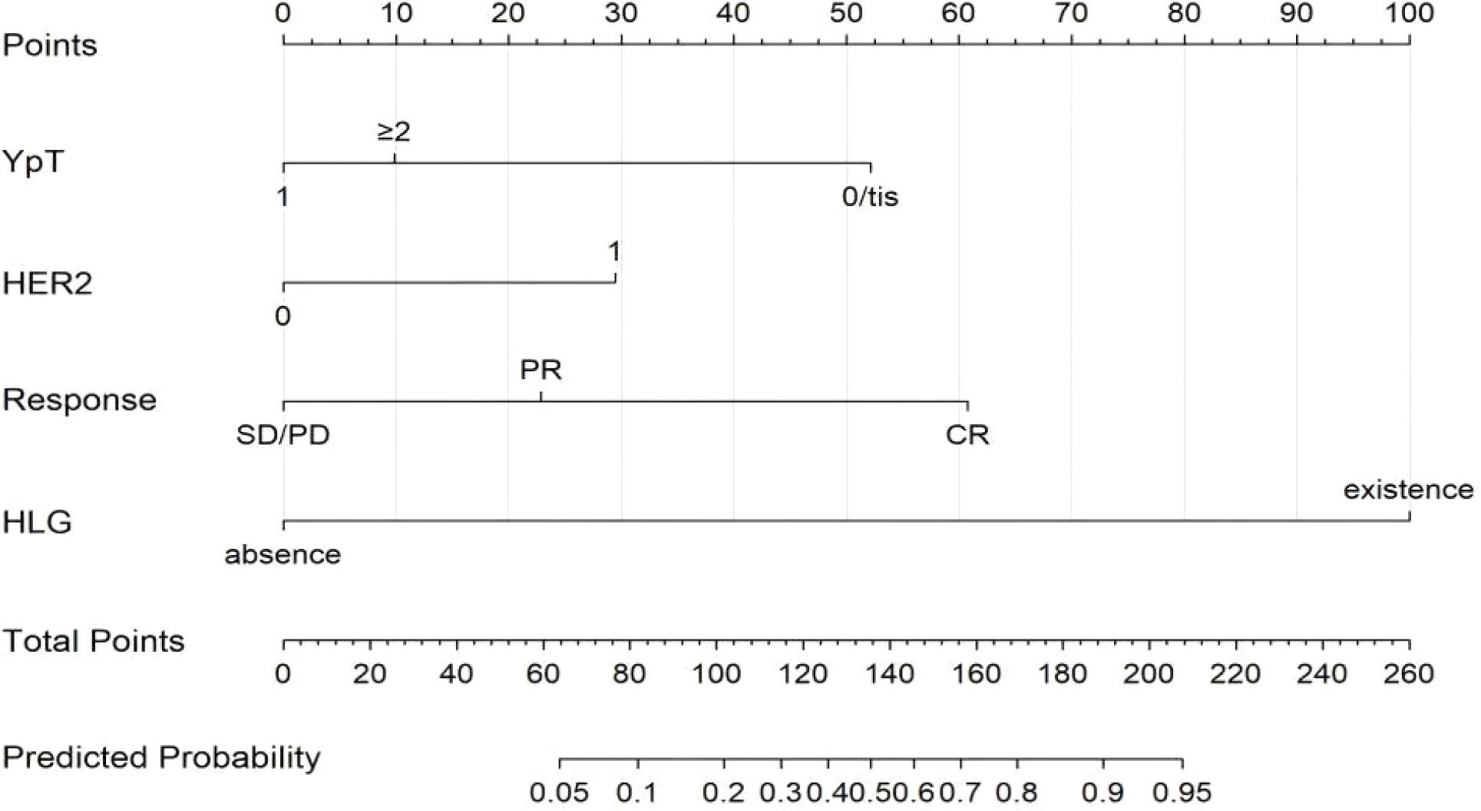
Nomogram to predict the individual probability of axillary nodal pCR after NAT. PR, Partial Response; SD, Stable Disease; PD, Progressive Disease; ypT, Pathological T stage after neoadjuvant therapy; HLG, Hilum of Lymph Gland.

**Figure 4:**
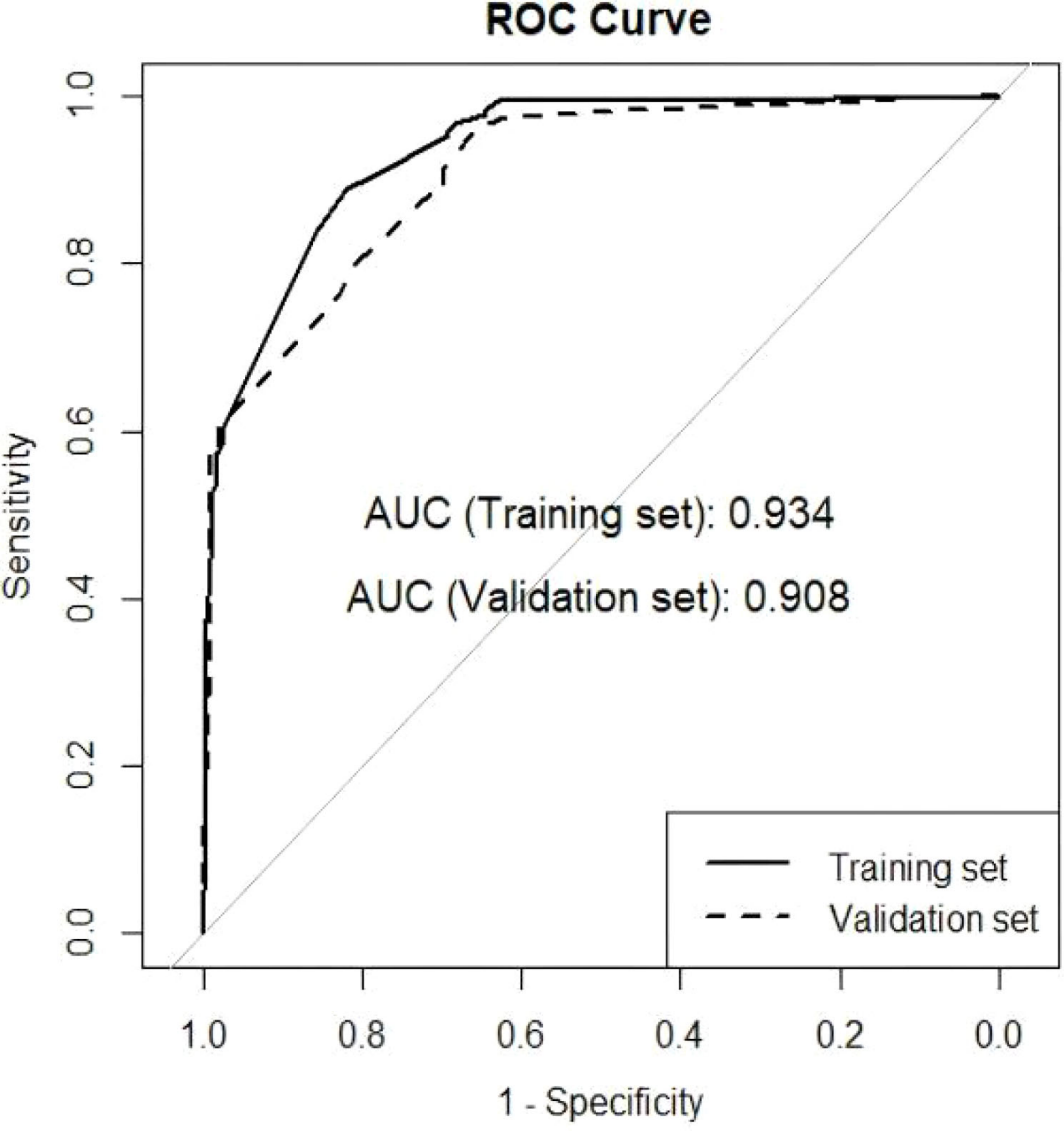
The receiver operating characteristic (ROC) curve of the nomogram, with an area under the curve (AUC) of 0.934 in the training cohort and an AUC of 0.908 in the validation cohort.

On the basis of the multivariate regression analysis, we included four meaningful variables significantly associated with axillary pCR and constructed a predictive nomogram (**Figure 3**). The total score was calculated for ypT stages, HER-2, the presence of the Hilum of the lymph gland on the US image after NAT, and the clinical response to NAT. These variables corresponded to a score on the axis of the quantity table, and the total score obtained by summing the individual scores. The probability of ALN pCR after NAT was then determined by projecting the total score onto the probability axis. In the ROC analysis, clinicopathological and imaging features resulted in an AUC of 0.934 (95% CI 0.913-0.960) in the training set and an AUC of 0.908 (95% CI: 0.867-0.950) in the validation set (**Figure 4**). The DeLong test showed no statistically significant difference between the AUCs of the training and validation sets (p = 0.272), confirming the model's generalizability. The prediction model details are shown in **Tables 5**, **6**, indicating that the nomogram had good discriminatory ability.

**Table 5:** Performance of the prediction model.

Metrics	Clinical prediction model		*P* value
	Training set	Validation set	
Accuracy (95% CI)	0.852 (0.817,0.886)	0.805 (0.750,0.861)	<0.001
AUC (95% CI)	0.934 (0.913,0.960)	0.908 (0.867,0.950)	0.272
Sensitivity (95% CI)	0.820 (0.770,0.870)	0.808 (0.724,0.884)	<0.001
Specificity (95% CI)	0.891 (0.842,0.933)	0.802 (0.721,0.886)	<0.001
PPV(%)(95% CI)	0.899 (0.860,0.939)	0.833 (0.758,0.903)	<0.001
NPV(%)(95% CI)	0.807 (0.752,0.859)	0.774 (0.681,0.861)	<0.001

AUC, Area Under the Curve; CI, Confidence Interval; PPV, Positive Predictive Value; NPV, Negative Predictive Value.

**Table 6:** Performance and Hosmer-Lemeshow of the prediction model.

Dataset Cohort	N	Negative LNs	AUC	95%CI	Hosmer-Lemeshow		
					X-squared	DF	P value
Training set	420	228	0.934	0.913-0.960	1.455	2	0.483
Validation set	180	99	0.908	0.867-0.950	2.89	2	0.235

### Nomogram validation

**Figure 5:**
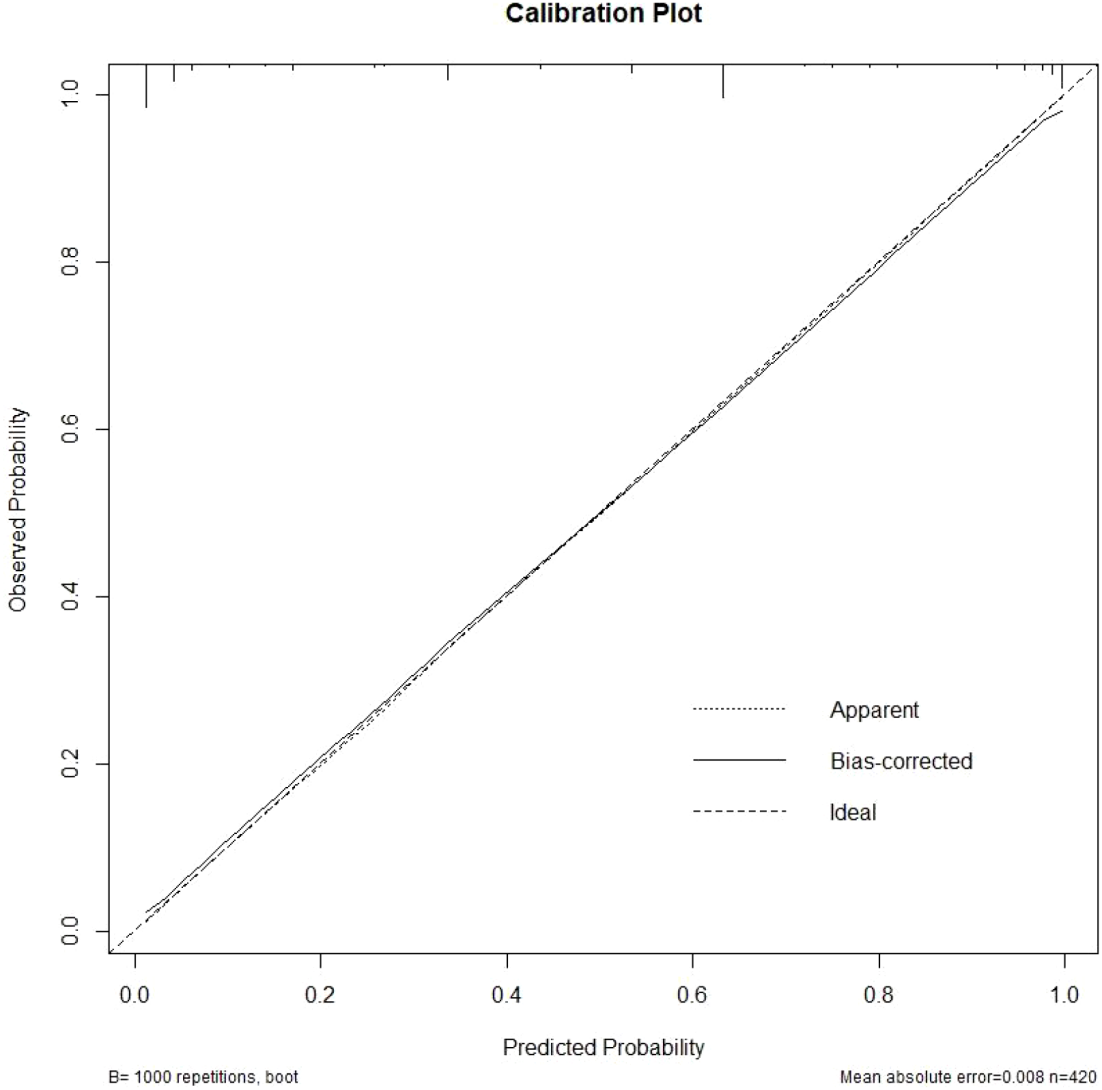
Calibration curves of the nomogram in the training cohort.

Next, we evaluated the outcome of the model on the basis of the training set calibration curve (**Figure 5**). The predicted results of the nomogram model have a good correlation with the actual state of ALN pCR. These results further confirm the reliability of the nomogram model established on the basis of the combination of clinical characteristics and imaging features. The calibration plot shows good consistency between the predicted performance of ALN-pCR and the actual results of ALN-pCR cases in the validation cohort (Hosmer-NSLN GOF test; P = 0.235). To better apply the prediction model, we determined a cutoff value on the basis of the Youden index in the training cohort (the prediction probability of ALN turning negative = 0.520). From this cutoff, we calculated the accuracy, sensitivity, and specificity of both patient groups, as shown in **Tables 5**, **6**.

## Discussion

In this study, a model was established based on imaging and clinical pathological variables to predict the ALN status after NAT. We identified four variables as independent predictors of ALN pCR, which were ypT, HER-2, the presence of the Hilum of the lymph gland on US after NAT, and the efficacy evaluation of NAT. Analysis of the training and validation sets revealed that the model can predict ALN pCR after NAT. Moreover, the calibration curve revealed that the prediction model has a relatively satisfactory calibration effect. In conclusion, our results show that the newly constructed nomogram is a reliable, relatively objective model with excellent stability, to make better predictions of ALN pCR, provides important guidance for surgeons in making surgical decisions, and can prevent unnecessary axillary surgery.

The clinical application value of this nomogram is multifaceted. First, it can be integrated into the preoperative workflow through radiologist-clinician collaboration: after post-NAT imaging, radiologists can assess the four predictors (ypT stage, HER2 status, fatty hilum presence, and tumor response), and clinicians can calculate the nomogram score to estimate the probability of axillary pCR. Patients with high predicted probabilities of pCR may be candidates for de-escalated axillary surgery (e.g., sentinel lymph node biopsy alone), while those with low predicted probabilities may proceed directly to ALND. Second, compared with existing models, our nomogram demonstrated superior discriminatory performance. Caudle et al. (2016) reported an AUC of 0.72 for predicting axillary pCR using clinical factors alone; our multimodal approach achieved an AUC of 0.934. Kim et al. (2015) achieved an AUC of 0.82 using clinicopathological variables without post-NAT imaging features. The integration of post-NAT ultrasound findings, particularly the fatty hilum status, contributes to our model's improved performance. Third, regarding standardization across institutions, the imaging criteria used in our model (tumor size on MRI and fatty hilum presence on US) are based on widely accepted radiological standards and can be applied in most centers with breast imaging expertise.

As the most widely used imaging examination, US still cannot accurately determine the ALN status (29, 31). This study integrated clinical pathology, ultrasound, and MRI features to establish a model that can predict ALN pCR, improving prediction efficiency. Our study predicted the ALN status by diagnosis and changes in imaging findings before and after NAT. In previous studies on US, MG, MRI, and CT, US and MRI were the best methods for predicting ALN status (32, 33), whereas MG could not be used to accurately predict ALN status (34, 35). Our findings are similar to previous studies. In our study, we found that the presence of the Hilum of the lymph gland on US after NAT was an independent factor of ALN status.

In this study, HER-2 status was shown to be an independent factor of ALN status (p <0.001). Molecular subtypes are important indicators for predicting the response of BC patients to NAT. It is also meaningful as a predictor in the model. Previous studies have established several nomograms to predict the ALN pCR after NAT (21–24). Previous researchers have included clinical factors, including ER, PR, HER2, and Ki67, as independent predictive factors. This study revealed a greater probability of ALN pCR after NAT in HER2-positive BC patients, and these findings are also consistent with the outcomes of this research. Our findings suggest that IHC has significant value in the prediction of ALN pCR after NAT and deserves the attention of clinicians.

Furthermore, we found that changes in the primary lesions of breast cancer patients can also be used as indicators of ALN status. We found that both ypT and MR assessment efficacy were independent factors of ALN pCR after NAT. Previous findings focused on the size of the primary lesion as an index in predicting ALN status (36–38). However, NAT usually reduces the burden of BC. In this study, the probability of achieving ALN pCR reached 87.3% in ypT0/tis patients. We included changes in MR lesions before and after neoadjuvant treatment and divided these patients into CR, PR, and SD/PD groups according to the RECIST criteria. We found that the probability of achieving ALN pCR was 91.5% if the primary breast lesion achieved CR and 67.4% if CR was not achieved. These findings suggest that it is reliable to estimate ALN pCR by determining whether the primary lesion has reached CR. Only a few studies have combined ALN ultrasound images, the response of breast lesions to NAT on MR images, and clinicopathological features. Our study allowed for a comprehensive evaluation of the relationships between these parameters and ALN pCR.

The developed model in our study showed better predictive performance compared to previous studies, but this study also has certain limitations. First, this study was a single-center retrospective study, which may introduce selection bias and limit the generalizability of our findings to other populations. External validation using data from other centers with diverse patient populations is warranted to confirm the model's applicability. Second, patients with incomplete imaging or clinical data (n=47) were excluded, which may have introduced selection bias. The impact of missing data on model performance was not formally assessed through sensitivity analyses. Third, although we used a standardized imaging protocol, inter-institutional variability in US and MRI acquisition and interpretation may affect the reproducibility of our model. Fourth, radiomics features were not incorporated into this model, which may provide additional predictive information. Finally, the relatively long study period (2011–2024) may introduce heterogeneity due to evolving NAT regimens and imaging techniques. Future studies addressing these limitations are warranted to validate the broader utility of our nomogram model.

A new prediction nomogram based on ultrasound, MRI, and clinicopathological features can evaluate the ALN status in cN+ BC patients after NAT. This model has the potential to become a practical, non-invasive clinical prediction tool. There is also a certain false-negative rate when SNB is used after NAT, and this model can improve the accuracy of ALN pCR prediction. Further clinical examination and improvement of the model are expected to result in extensive clinical promotion to assist surgeons in clinical decision-making and avoid unnecessary ALND.

## Data Availability

The original contributions presented in the study are included in the article/supplementary material. Further inquiries can be directed to the corresponding author.
